# Taxonomic assessment of two wild house mouse subspecies using whole-genome sequencing

**DOI:** 10.1038/s41598-022-25420-x

**Published:** 2022-12-02

**Authors:** Raman Akinyanju Lawal, Verity L. Mathis, Mary E. Barter, Jeremy R. Charette, Alexis Garretson, Beth L. Dumont

**Affiliations:** 1 The Jackson Laboratory, 600 Main Street, Bar Harbor, 04609 USA; 2 Florida Museum of Natural History, University of Florida, 1659 Museum Road, Gainesville, FL 32611 USA; 3 Graduate School of Biomedical Sciences, Tufts University, 136 Harrison Ave, Boston, MA 02111 USA

**Keywords:** Evolutionary genetics, Population genetics

## Abstract

The house mouse species complex (*Mus musculus*) is comprised of three primary subspecies. A large number of secondary subspecies have also been suggested on the basis of divergent morphology and molecular variation at limited numbers of markers. While the phylogenetic relationships among the primary *M. musculus* subspecies are well-defined, relationships among secondary subspecies and between secondary and primary subspecies remain less clear. Here, we integrate de novo genome sequencing of museum-stored specimens of house mice from one secondary subspecies (*M. m. bactrianus*) and publicly available genome sequences of house mice previously characterized as *M. m. helgolandicus,* with whole genome sequences from diverse representatives of the three primary house mouse subspecies. We show that mice assigned to the secondary *M. m. bactrianus* and *M. m. helgolandicus* subspecies are not genetically differentiated from *M. m. castaneus* and *M. m. domesticus*, respectively. Overall, our work suggests that the *M. m. bactrianus* and *M. m. helgolandicus* subspecies are not well-justified taxonomic entities, emphasizing the importance of leveraging whole-genome sequence data to inform subspecies designations. Additionally, our investigation provides tailored experimental procedures for generating whole genome sequences from air-dried mouse skins, along with key genomic resources to inform future genomic studies of wild mouse diversity.

## Introduction

House mice (*M. musculus*) are the premiere mammalian model system for biomedical research and an important natural model system for ecological and evolutionary studies^1,2^. House mice emerged from an ancestral population in the Indian subcontinent less than 3 million years ago^3,4^ and subsequently expanded out of this ancestral region, giving rise to three primary subspecies^5–8^. *M. m. domesticus* (DOM) is native to Western Europe, *M. m. musculus* (MUS) is present across Eastern Europe and Siberia, and *M. m. castaneus* (CAS) extends across South and Southeast Asia. Aided by human dispersal in recent history, house mice have subsequently expanded their footprint outside of these native ranges, colonizing all major continents except Antarctica and invading many remote oceanic islands.

Beyond these three primary subspecies, a number of secondary house mouse subspecies have been suggested on the basis of distinct morphology^9–11^ and surveys of limited numbers of molecular markers^6,8,11–15^. For example, mice from Yemen and Madagascar have been assigned to *M. m. gentilulus* due to their small body size and distinct mitochondrial lineage^12,13^. Mice from Heligoland, a small German archipelago island in the North Sea, have been characterized as *M. m. helgolandicus* on the basis of their unique skull morphology, distinct mitochondrial D-loop haplotype, and allelic variation at four nuclear loci^11,16^. Similarly, a white belly coat color phenotype^17^ and mitochondrial sequence analysis have supported the assignment of house mice from the Indo-Iranian valley to the subspecies *M. m. bactrianus*^15,18^. *M. m. musculus* and *M. m. castaneus* naturally hybridize where their ranges overlap in Japan, and these hybrids have been designated as a distinct subspecies, *M. m. molossinus*^19^. At least six other secondary subspecies of *M. musculus* have been named, including: *M. m. albula, M. m. brevirostris, M. m. homourus, M. m. isatissus, M. m. wagneri,* and *M. m. gansuensis*^20^.

Prior studies have leveraged powerful genomic approaches to investigate the evolutionary relationships between the three primary house mouse subspecies, establishing a sister relationship between *M. m. castaneus* and *M. m. musculus*^21–25^. In contrast, the phylogenetic relationships among secondary house mouse subspecies, including their relationships to the primary house mouse subspecies, remain poorly understood. Currently, all secondary subspecies assignments are informed by sparse molecular data, begging the question of whether distinct subspecies labels are truly warranted. Indeed, some subspecies designations have been proposed based on only mitochondrial genetic markers (e.g.,^13^), despite knowledge that mitochondrial inferences provide a strict matrilineal reconstruction of subspecies relationships and may be misleading in the face of sex differences in dispersal or evolutionary history^26^. Subspecies assignments relying on genotypes at limited numbers of nuclear loci are similarly problematic, as the chance sampling of population-private alleles can be misinterpreted as evidence supporting new subspecies designations. Overall, the legitimacy of specific secondary house mouse subspecies remains debatable, with genome-scale investigations standing to provide an ultimate resolution to their taxonomic status.

The democratization of DNA sequencing has lowered the cost barrier to genomic investigations across biological disciplines, including phylogenetics and systematics. As a result, sample availability and sample collection have emerged as comparatively greater challenges for many studies. Although house mice are ubiquitous across the globe, many secondary subspecies inhabit small, isolated regions that are not readily accessible. Procedural barriers, including securing necessary permits and customs paperwork, also pose formidable challenges to wild sample collection. As an alternative to live sample collection, many natural history museums harbor large holdings of air-dried animal skins that could provide necessary tissue material for ‘omics studies. However, exposure to air, light, and chemicals during long-term storage can lead to extensive DNA degradation and damage, posing technical challenges for DNA isolation, amplification, and sequencing from preserved tissues^27,28^. Protocols for DNA extraction have been developed for historical mammalian specimens (e.g.,^29^), and both genotyping approaches and low coverage whole-genome sequencing methods have been adapted for use on low-quality, archived biospecimens (reviewed in^28,30^). However, to our knowledge, no moderate- to high-coverage whole-genome sequences (WGS) have been generated from archived non-human mammalian tissues, raising uncertainties about the technical feasibility of this approach (but see^31^ for exome sequencing and^32^ for low coverage WGS of mammalian species).

Here, we combine published WGS from wild mice^33–35^ with de novo genome sequencing of a strategic set of museum-preserved specimens to address the validity of two secondary house mouse subspecies designations—*M. m. bactrianus* and *M. m. helgolandicus*. These subspecies labels have been assigned on the basis of subtle morphological differences from the primary house mouse subspecies and divergence at both mitochondrial and limited numbers of nuclear DNA markers^11,15,18^. Our investigations offer a conclusive resolution to the taxonomic status of mice from these subspecies, arguing that these taxonomic assignments are not well justified based on genomic data. Further, our work provides a crucial proof-of-concept demonstration that moderate-coverage whole genome sequences can be readily obtained from archived mammalian tissue.

## Results and discussion

### Whole-genome sequencing of museum samples of wild-caught mice from Pakistan

**Figure 1:**
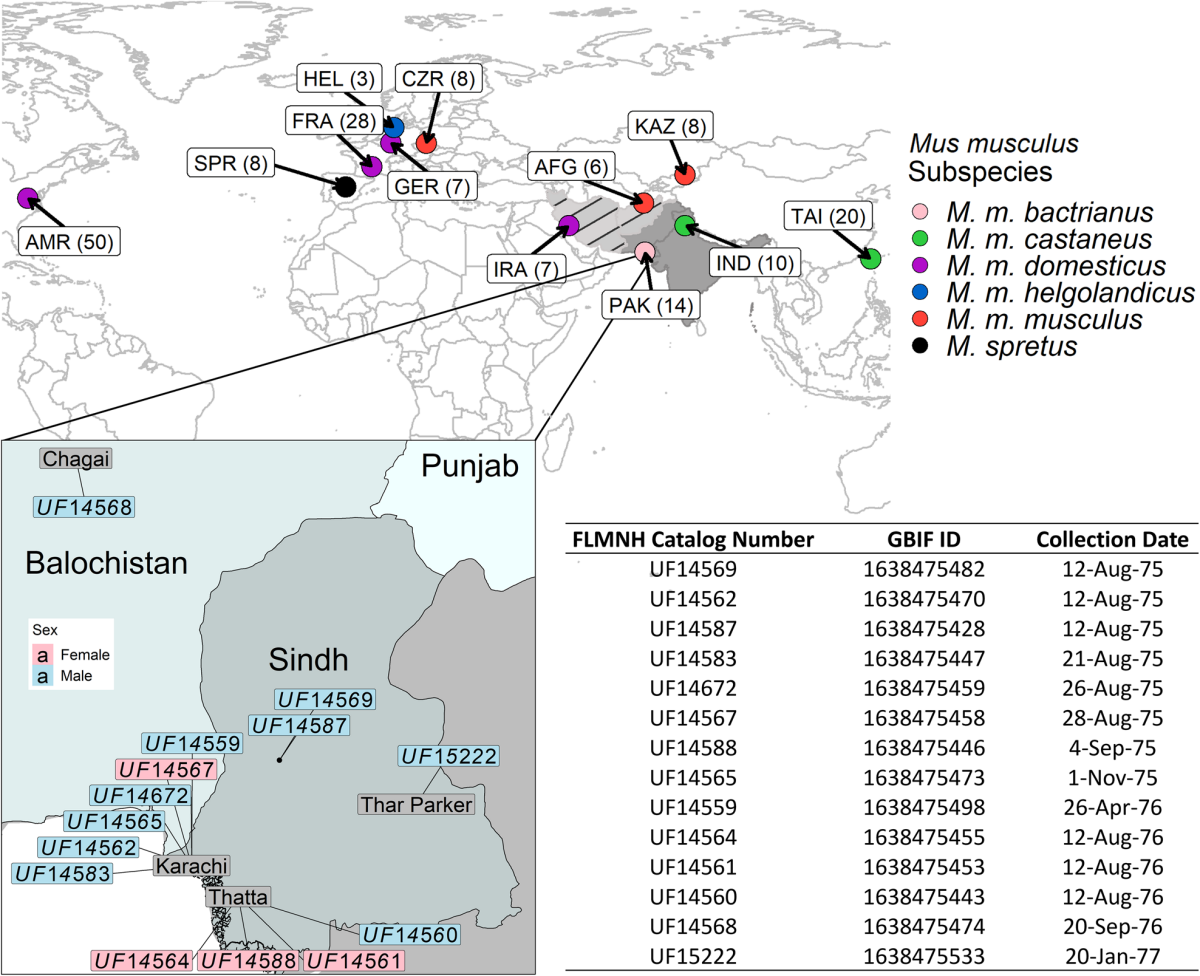
Map showing the approximate geographic sampling locations of house mouse populations profiled by whole-genome sequencing. The house mouse ancestral region extends from India “IND” and Pakistan “PAK” (dark grey area) and may include the broader region from Iran “IRA” and Afghanistan “AFG” (light grey area with black markings). All genome sequences except for PAK were retrieved from public databases^33–35^. The additional sequences include populations from America (AMR), France (FRA), Germany (GER), Heligoland (HEL), Kazakhstan (KAZ), and Czech Republic (CZR), Taiwan (TAI), and *M. spretus* from Spain (SPR). Each population sample sizes are indicated in the bracket. The enlarged plot inset shows the locations of PAK samples collected across four counties in the Sindh region of Pakistan. Florida Museum of Natural History (FMNH) Catalog numbers, Global Biodiversity Information Facility (GBIF) identifiers^41^, and collection dates for each PAK sample are presented in the table.

We obtained air-dried skin snips from 14 ~46-year-old museum-preserved specimens of *M. m. bactrianus* initially collected across several counties in Pakistan (PAK) and maintained by the Florida Museum of Natural History (Fig. 1). This geographic sample region overlaps with the presumed ancestral homeland of house mice^6^. We adapted an existing protocol for DNA extraction from museum-preserved mammalian pelts^29^, isolating between 0.06 and 1 μg of fragmented DNA (< 500 bp) per specimen. DNA samples were processed for Illumina library preparation and whole-genome sequenced to 17× – 45× coverage using 100 bp paired-end reads (Supplementary Fig. S1a). On average, ~ 99% of sequenced reads from each of these mouse genomes were successfully mapped to the mm10 reference (Supplementary Fig. S1b), suggesting little exogenous contamination of our samples. This represents a dramatic improvement in mapping rate relative to other sequencing studies of archived biospecimens and ancient DNA (~ 20–75%)^28,32^. The proportion of bases supported by at least 5 reads ranges from 66 to 96% across samples (Supplementary Fig. S1c), indicating excellent genomic coverage.

Ancient and museum-stored specimens are known to accumulate C → T and A → G mutations due to post-mortem DNA deamination^36^. This spontaneous damage can lead to sequence biases if not properly accounted for. We determine that, across the PAK genomes, the deamination rate for nucleotide misincorporation is ~ 0.1%, with nearly all damage confined to the first and last 5 bp of a given read (see Methods; Supplementary Fig. S2). By comparison, the frequency of post-mortem DNA damage in a sample of ancient (~ 13 k years old) stickleback fish was 30%^37^ and is typically around 50% for Neanderthal DNA^38^. Although there is minimal post-mortem DNA damage in our sequenced PAK genomes, we took the conservative approach of hard clipping the terminal 5 bp on both the 5′ and 3′ ends of every read to eliminate potential artifacts (Supplementary Fig. S1d).

After applying these quality control steps, performing variant calling, and imposing basic hard filters to eliminate low-quality variant calls (see Methods), we identified a total of 81,338,251 autosomal SNPs and 8,802,753 short indels across the 14 PAK genomes. This corresponds to ~ 1 variant every 30 bases relative to the mm10 reference genome.

### Re-evaluating phylogenetic relationships among *M. musculus* subspecies

We combined the 14 PAK genome sequences with 3 previously published genome sequences of *M*. *m. helgolandicus* from Heligoland (HEL) and 152 publicly available whole-genome sequences from wild-caught mice from multiple populations of *M. m. castaneus* (CAS), *M. m. musculus* (MUS), and *M. m. domesticus* (DOM)^33–35^. Using this comprehensive set of 169 whole genome sequences, we evaluated the genetic relationships among these five putative subspecies using a multi-pronged approach.

**Figure 2:**
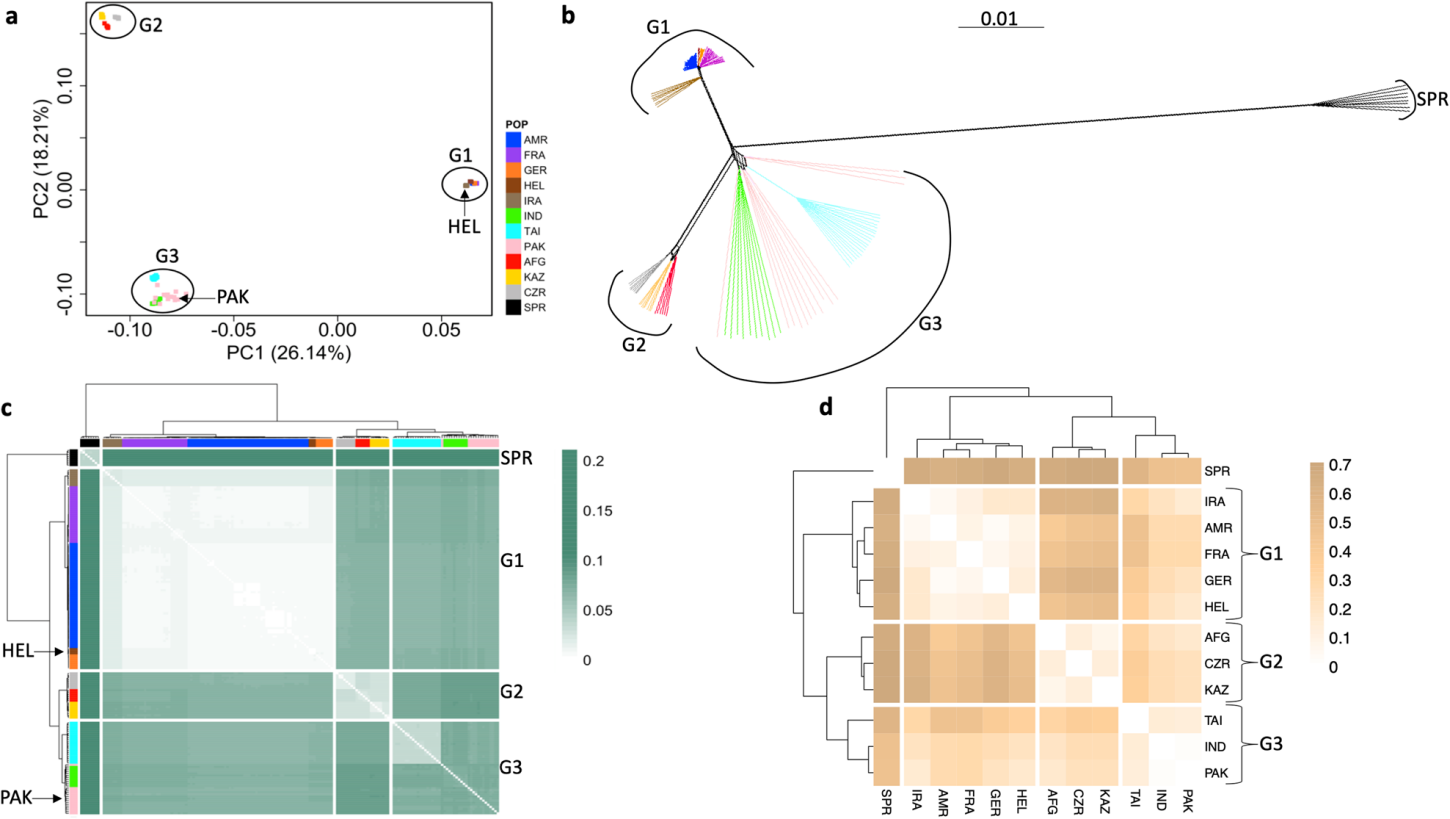
Genetic relationships among five putative *M. musculus* subspecies reveal three taxonomic groups (G1–G3). Genetic relationships were assessed via (**a**) principal component analysis, (**b**) a phylogenetic tree constructed from a pairwise distance matrix, (**c**) allele sharing distance, and (**d**) co-ancestry based on F_ST_. The color legend on the right side of panel (**a**) is also applicable to panels (**b**) and (**c**). G1 groups populations belonging to the DOM subspecies: America (AMR), France (FRA), Germany (GER), Heligoland (HEL), and Iran (IRA). G2 groups populations of MUS: Afghanistan (AFG), Kazakhstan (KAZ), and Czech Republic (CZR). G3 groups CAS populations: India (IND), Taiwan (TAI), and Pakistan (PAK). *M. spretus* (SPR) is used as an outgroup.

First, we conducted a principal component analysis to visualize genetic similarities across samples (Fig. 2a). The first two principal components, accounting for 26.14% and 18.21% of the total variance, reveal just three discrete clusters (G1–G3) corresponding to the primary DOM, MUS, and CAS subspecies. Mice from the HEL population group within DOM, while PAK is nearly indistinguishable from the CAS populations (Fig. 2a). Additional approaches based on average pairwise distance among individuals (Fig. 2b), allele sharing distance (Fig. 2c), and co-ancestry (Fig. 2d) also support three discrete phylogenetic units aligning to the three primary *M. musculus* subspecies. These findings are in agreement with a recent study that similarly found support for only three primary *M. musculus* clades using an independent set of whole genome sequences from mice across the globe^25^.

**Figure 3:**
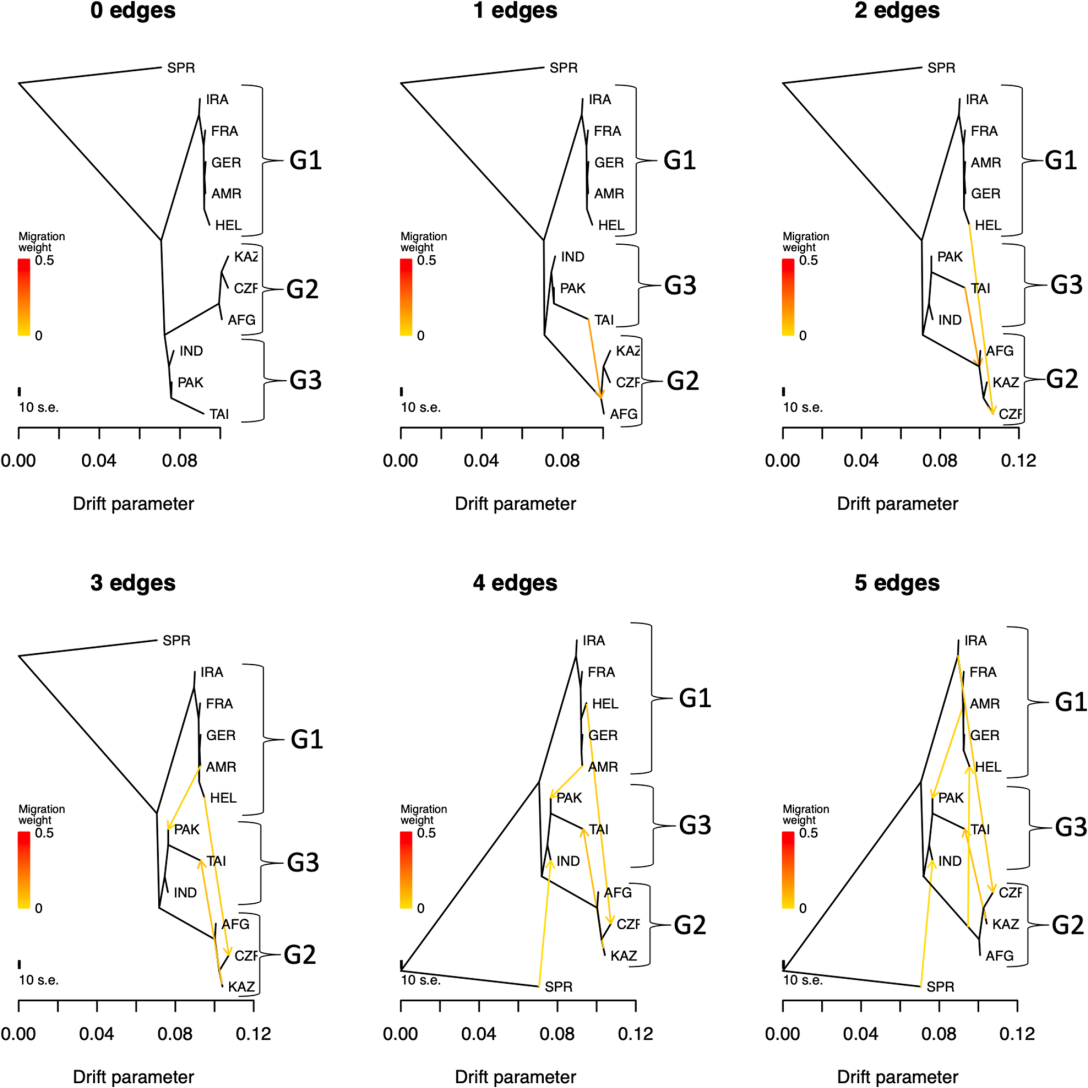
Phylogenetic relationships from TreeMix between house mouse populations. Treemix models the effect of different numbers of populations experiencing gene flow and different pairwise migration rates between populations. Zero edges correspond to the absence of gene flow. Under all considered scenarios, HEL is embedded within the taxonomic group G1 (*M. m. domesticus*) and PAK in G3 (corresponding to *M. m. castaneus* populations). *M. spretus* (SPR) was used as an outgroup.

Wild mice may experience substantial gene flow between populations and subspecies^39^, which could obscure subspecies relationships. To evaluate the possible influence of gene flow on our inferred subspecies groupings, we used TreeMix to assess the robustness of our findings under multiple distinct migration models^40^ (see Methods). We recovered identical genealogical relationships both in the absence of gene flow and under different migration scenarios (*p* < 1 × 10^–300^; Fig. 3). Importantly, no tested migration model altered the composition of the three core subspecies groups or offered support for the *M. m. helgolandicus* or *M. m. bactrianus* subspecies designations.

We find no compelling genetic evidence to justify a unique subspecies designation for mice from HEL or PAK. These conclusions contradict earlier findings based on limited numbers of genomic markers^11^ or single loci^15^, underscoring the power and importance of leveraging whole-genome data to inform subspecies designations.

**Figure 4:**
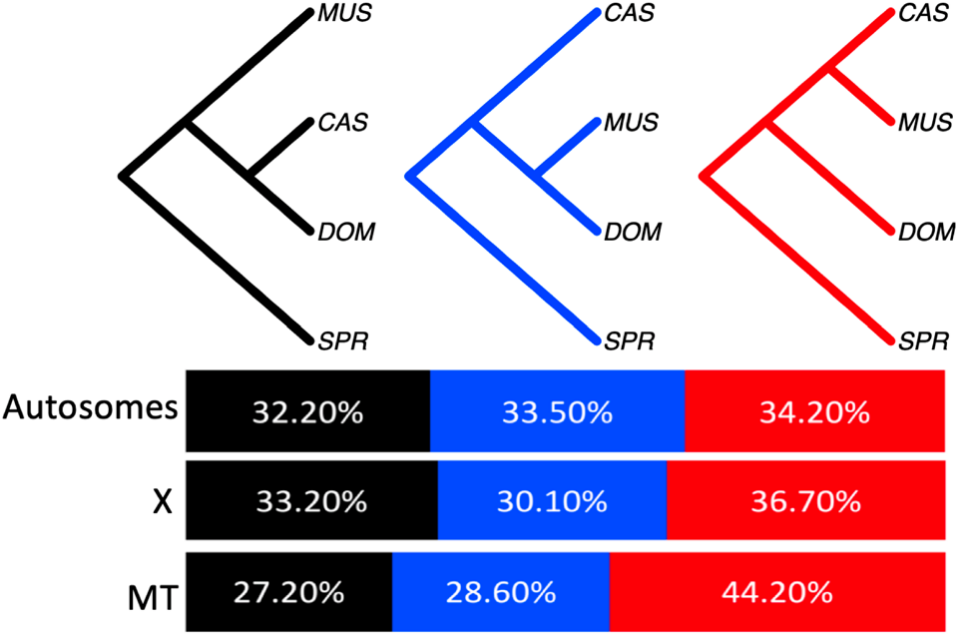
The percentage of the autosomal, X, and mitochondrial (MT) genome supporting each of the three possible topological relationships relating the three primary house mouse subspecies. Percentages correspond to the representation of each topology across 13,122 genomic regions. *M. m. castaneus* (CAS), *M. m. musculus* (MUS), *M. m. domesticus* (DOM), and outgroup *M. spretus* (SPR).

Our investigations suggest that PAK and HEL are populations of *M. m. castaneus* and *M. m. domesticus*, respectively. Combining these populations with their modified subspecies groupings, we next partitioned the genome into 13,122 blocks, constructed phylogenetic trees from each partition, and estimated the percentage of the genome supporting each of the three possible topologies relating the three primary *M. musculus* subspecies. Overall, the topology placing CAS and MUS as sister is the most abundant in the genome, capturing a total weight of 34.20% (autosomes), 36.70% (X chromosome), and 44.20% (MT) (Fig. 4). This finding validates prior conclusions about house mouse subspecies phylogenetic relationships based on representative inbred strain genomes^21,22^ and smaller samples of wild mice^23,25^. Notably, our use of a broader set of wild house mouse genomes sampled from the presumed ancestral region offers a more comprehensive survey of ancestral house mouse diversity, leading to increased power to detect alleles that are still segregating across multiple subspecies due to incomplete lineage sorting. In turn, this improved ancestral sampling is expected to allow more accurate estimates of the extent of phylogenetic discordance across the *M. musculus* genome, as well as the proportional representation of each topology.

## Conclusions

Using both new and published whole-genome sequences from diverse wild house mice, we addressed support for two secondary *M. musculus* subspecies assignments: *M. m. bactrianus* and *M. m. helgolandicus.* We showed that mice from Pakistan previously assigned to *M. m. bactrianus* are genetically indistinguishable from *M. m. castaneus* mice. Similarly, mice assigned to the *M. m. helgolandicus* subspecies exhibit no meaningful genome-wide pattern of divergence from *M. m. domesticus*. While these subspecies may harbor distinct morphological adaptations^11^, the adoption of a strict genetic species concept argues that *M. m. bactrianus* and *M. m. helgolandicus* are not well-justified taxonomic groups. Instead, mice previously assigned to these subspecies appear to capture population-level genetic diversity within the primary house mouse subspecies. Our work motivates additional genomic investigations into whether other secondary house mouse subspecies designations are warranted.

In addition to providing novel insights into house mouse taxonomy, our work yields newly sequenced house mouse genomes that will serve as key genetic resources for future investigations into wild mouse demographic history and diversity. In particular, genome sequencing of wild mice sampled from the cradle of *M. musculus* evolution will enable studies of the impact of ancestral variation on contemporary patterns of global wild mouse diversity. Further, our work has established the feasibility of whole genome sequencing of archived mammalian tissue and represents an important advance for the emerging discipline of museomics. Broader application of this methodology to additional museum samples will offer a facile approach for strategically expanding genomic catalogs of wild mouse diversity, and potentially population genomic and reference genome sequencing of other mammalian species.

## Materials and methods

### Museum sample collection

We destructively sampled the skins of 14 M*. m. bactrianus* mice housed in the Florida Museum of Natural History Mammalogy Collection (http://specifyportal.flmnh.ufl.edu/mammals/)*.* These specimens were collected and preserved between 1975 and 1977 across four counties in Pakistan ‘PAK’ (see Fig. 1 inset). Further details can be found in the GBIF.org (25 April 2022) GBIF occurrence download (https://doi.org/10.15468/dl.xuksm3)^41^. Skin snips were obtained by removing a small (approx. 5 × 5 mm) section of skin from the ventral side of the study skin, sterilizing instruments in between samples.

### DNA extraction protocol for museum-stored samples

DNA was isolated from desiccated skin samples following a previously published protocol^29^ with minor modifications. Briefly, the skin samples were scraped with a sterilized scalpel to remove possible contaminants. Samples were transferred to a 2 ml tube, washed three times with sterile water, three times with 70% ethanol, three times with sterile water, and then cut into small pieces. Samples were hydrated before digestion by incubating for 24 h in 1 mL of TE (10 mM Tris; 1 mM EDTA, pH 7.6), washing with 70% ethanol and sterile water, and hydrating again in TE solution for a further 24 h. Samples were digested in TNE solution (10 mM Tris HCl, pH 8; 400 mM NaCl; 2 mM EDTA, pH 8.0) plus SDS 1% and Proteinase K (0.58 mg/ml final conc.) at 55 °C for 24–36 h until the tissue was completely digested. The DNA was extracted with one volume of phenol:chloroform:isoamilic alcohol (25:24:1), rotated at 20 rpm for 10 min, and centrifuged for 10 min at 4000 rcf, after which the supernatant solution was transferred to another tube. The DNA was precipitated by adding two volumes of 100% ethanol, gently inverting the tube, and maintaining the solution at − 20 °C for 16 h. The samples were centrifuged for 2 min at 3000 rcf before discarding the ethanol and resuspending the pellet in 50 μL of TE.

DNA concentration and quality (size) were assessed using the Nanodrop 2000 spectrophotometer (Thermo Scientific), the Qubit 3.0 dsDNA BR Assay (Thermo Scientific), and the D5000 DNA ScreenTape Analysis Assay (Agilent Technologies). DNA fragment sizes ranged from 76 to 431 bp. Only samples with DNA concentration > 63 ng/μl were used for genome sequencing.

### Genomic DNA library preparation

Whole-genome libraries were constructed using the KAPA HyperPrep Kit (Roche Sequencing and Life Science) according to the manufacturer’s protocols. No fragmentation or sizing was done on the samples before proceeding with ligation of Illumina-specific barcoded adapters and PCR amplification. The quality and concentration of the libraries were assessed using the D5000 ScreenTape (Agilent Technologies) and the KAPA Library Quantification Kit (Roche Sequencing and Life Science) according to the manufacturers’ instructions.

Libraries were pooled and sequenced on the NovaSeq 6000 (Illumina) using the S4 Reagent Kit (Illumina) and100 bp paired-end reads. We targeted 30X coverage per sample, with the amount of generated data ranging from 33 to 112 Gb across samples (see Supplementary Fig. S3).

### Evaluating museum-stored DNA for post-mortem damage

The long-term storage of museum specimens is associated with DNA degradation by deamination, leading to an excessive accumulation of cytosine to uracil (read by sequencer as thymine) changes^36^. In downstream analyses, such post-mortem DNA damage can lead to biases and incorrect data interpretation. We evaluated the PAK genome sequences derived from the museum-stored samples using the Bayesian approach implemented in mapDamage 2.0, a program designed to track and quantify DNA damage patterns^42^. Specifically, we focused our attention on the unusual accumulation of C to T and A to G mutations at the 5′ and 3′ termini as they represent the signatures of post-mortem deamination. Across the 14 PAK samples, the frequency of post-mortem DNA damage was estimated to be no greater than 0.1% of bases in each genome (Supplementary Fig. S2) and error signals were restricted to the 5 bp within the 5′ and 3′ termini of reads. To eliminate potential biases and errors in our data, we trimmed the first and last 5 bases from each read using the “trimBam” option in “BamUtil”^43^.

### Additional wild mouse genome sequences

We retrieved 155 previously published genomes belonging to *M. m. domesticus* (America, AMR = 50, France, FRA = 28, Germany, GER = 7, Iran = 7), *M. m. helgolandicus* (HEL, n = 3), *M. m. castaneus* (India, IND = 10, Taiwan, TAI = 20), *M. m. musculus* (Afghanistan, AFG = 6; Czech Republic, CZR = 8; Kazakhstan, KAZ = 8), and *M. spretus* (Spain, SPR = 8)^33–35^.

### Sequence alignment and variant calling

For the newly sequenced 14 PAK samples, we trimmed Illumina adapters using *cutadapt*^44^. The clean reads were mapped to mm10 reference genome using the default parameters in BWA version 0.7.15^45^. Data from the 14 PAK samples were processed simultaneously with the 155 publicly available genome sequences to generate an integrated call set. We followed the standard Genome Analysis Toolkit (GATK; version 4.2) pipeline for subsequent pre-processing before variant calling^46,47^. We performed variant calling for each sample separately using the “-ERC GVCF” mode in the “HaplotypeCaller”. Samples were then jointly genotyped using the “GenotypeGVCFs” GATK function and trained with previously ascertained mouse variants^21^ using both the “VariantRecalibrator” and “ApplyVQSR” option of GATK. For the latter, the truth sensitivity level to initiate filtration was set to the default (i.e., 99). We filtered variants to exclude sites with missing alleles using VCFtools version 0.1.16^48^. All downstream analyses focus on autosomal bi-allelic single nucleotide variants.

### Analyses of population genetic structure

Principal component analysis was performed on all 169 wild mouse genomes using Plink version 2.0^49^. To construct a distance matrix tree, we first reformatted the variant file using “bcftools view file.vcf | bcftools query -f′%CHROM\t%POS[\t%TGT]\n′ | sed -e ′s/\./N/g'” with BCFtools^50^. We then used the python script “distMat.py” obtained from https://github.com/simonhmartin/genomics_general to generate the tree matrix^51^. The tree was viewed using SplitsTree version 4.17.0^52^. We assessed the robustness of this topology to gene flow using TreeMix version 1.13^40^, allowing 0–5 migrations between any population pair in our dataset.

To calculate the allele sharing distance, we used the default option of “asd” (https://github.com/szpiech/asd)^53^ and viewed the data using the R package “pheatmap”^54^. We estimated the co-ancestry based Fst using the python program “popgenWindows.py” accessed from https://github.com/simonhmartin/genomics_general^51^, and visualized results using the pheatmap R package^54^.

### Inference of the dominant subspecies topology

We summarized the relationships among samples by building phylogenetic trees across 13,122 unique genomic regions, each defined by a fixed window of 50 SNPs. Trees were built for each window using the script “phyml_sliding_windows.py” accessed from https://github.com/simonhmartin/genomics_general. The output from the tree was used as input and weight assigned to each topology using *Twisst—*Topology Weighting by Iterative Sampling of Sub-Trees—based on the following options:—method complete,—abortCutoff 1000—backupMethod fixed—iterations 400^55^. Topologies were viewed in R using the package “APE” version 5.5^56^.

## Supplementary Information

Supplementary Information.

## Data Availability

The raw fastq reads of the newly sequenced 14 wild house mice from Pakistan have been deposited in the NCBI Short Read Archive under the BioProject accession PRJNA851025. https://www.ncbi.nlm.nih.gov/sra/?term=PRJNA851025.
