# Supplementary material for: Taxonomic assessment of two wild house mouse subspecies using whole-genome sequencing
Source: Sci Rep. 2022 Dec 2;12:20866. doi: 10.1038/s41598-022-25420-x (PMC9718808; doi:10.1038/s41598-022-25420-x)
Supplement: Supplementary file 1 — Supplementary Information. [file 41598_2022_25420_MOESM1_ESM.pdf]

Taxonomic assessment of two wild house mouse subspecies using whole genome sequencing

Raman Akinyanju Lawal[1*], Verity L. Mathis[2], Mary E. Barter[1], Jeremy R. Charette[1], Alexis Garretson[1,3], and Beth L. Dumont[1,3*]

[1]The Jackson Laboratory, 600 Main Street, 04609. Bar Harbor, USA.
[2]Florida Museum of Natural History, University of Florida, 1659 Museum Road, Gainesville FL 32611, USA.
[3]Tufts University, Graduate School of Biomedical Sciences, 136 Harrison Ave, Boston, MA, 02111, USA

[*]Correspondence: RAL (raman.lawal@jax.org) and BLD (beth.dumont@jax.org)

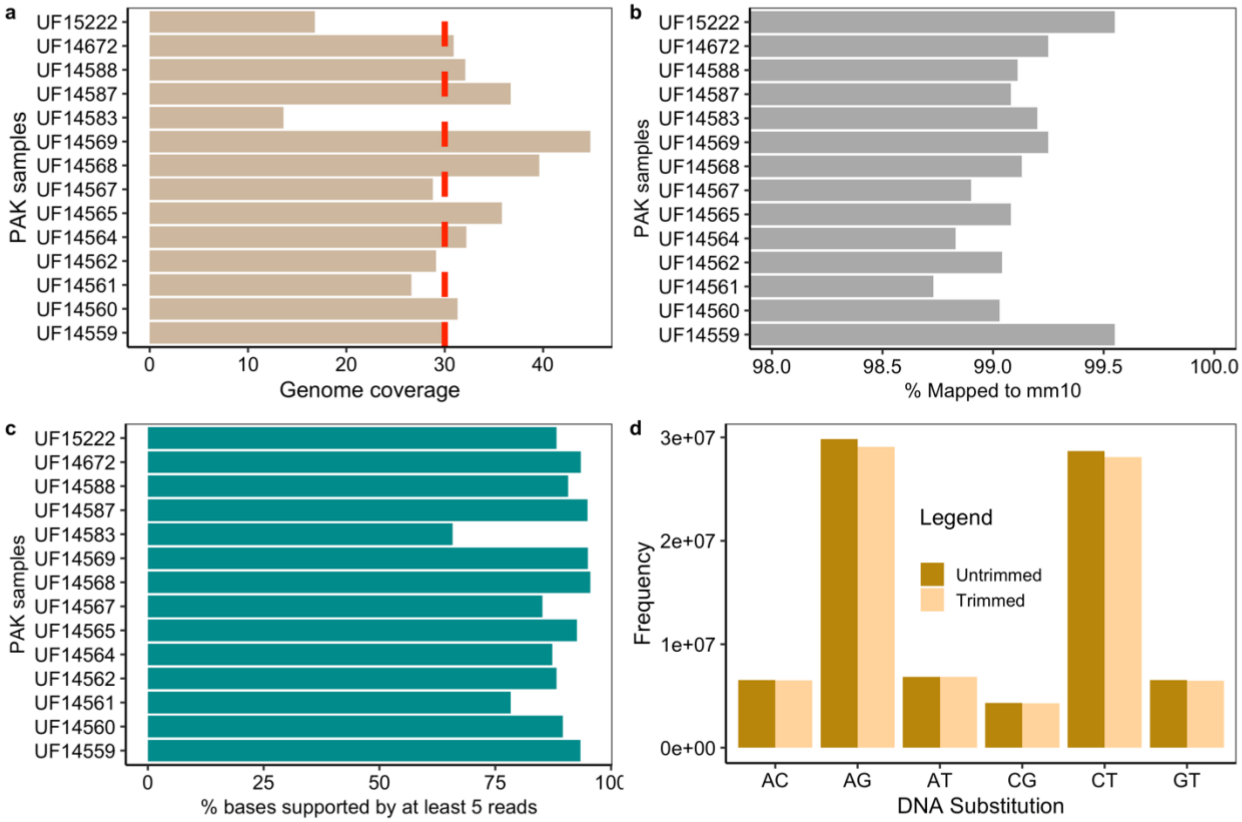

Supplementary Figure S1: Sequence and mapping statistics for 14 PAK house mouse genomes. (**a**) We targeted an average of 30x coverage per sample (red line), with actual coverage values ranging from 17-45x. Bar charts showing (**b**) the percentage of sequenced reads mapping to the mm10 reference genome per sample and (**c**) the percentage of bases supported by at least 5 reads. (**d**) DNA substitution rates before and after trimming the 5bp at the 3' and 5' termini of each read.

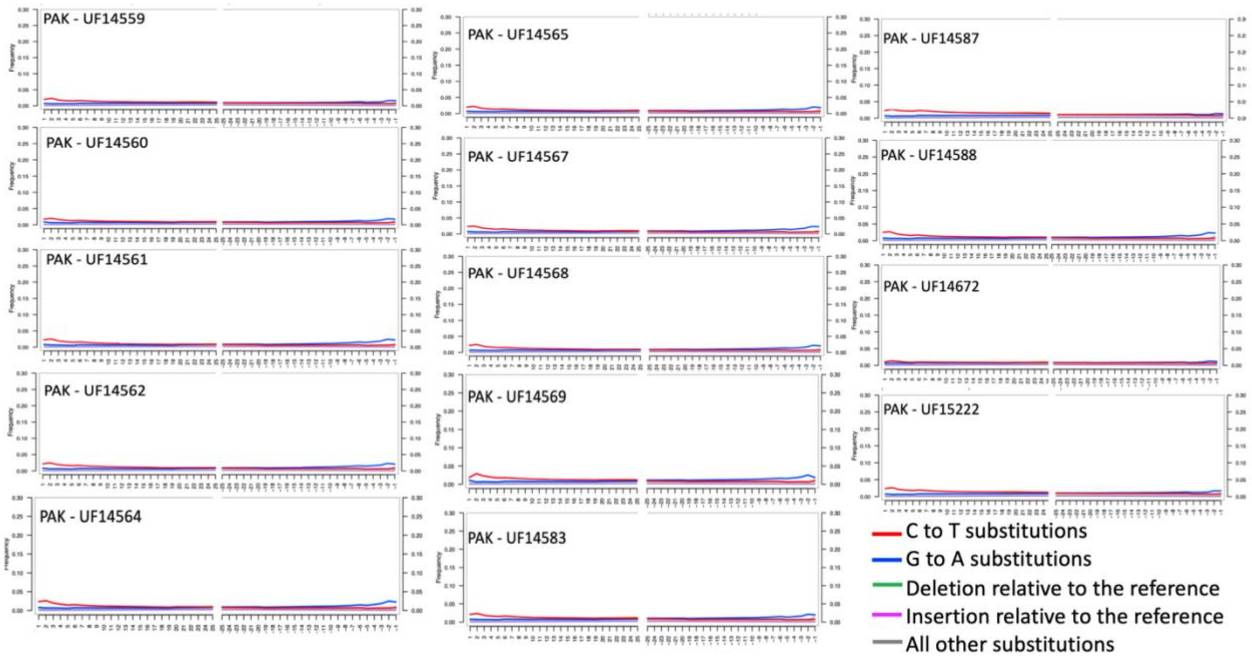

Supplementary Figure S2: The distribution of DNA misincorporation errors at both the 5´ and 3´ termini of sequenced reads from PAK house mice. The y-axis shows the frequency of post-mortem DNA damage relative to the mm10 reference genome. Post-mortem DNA damage is expected to lead to an excess accumulation of C>T and G>A substitutions.

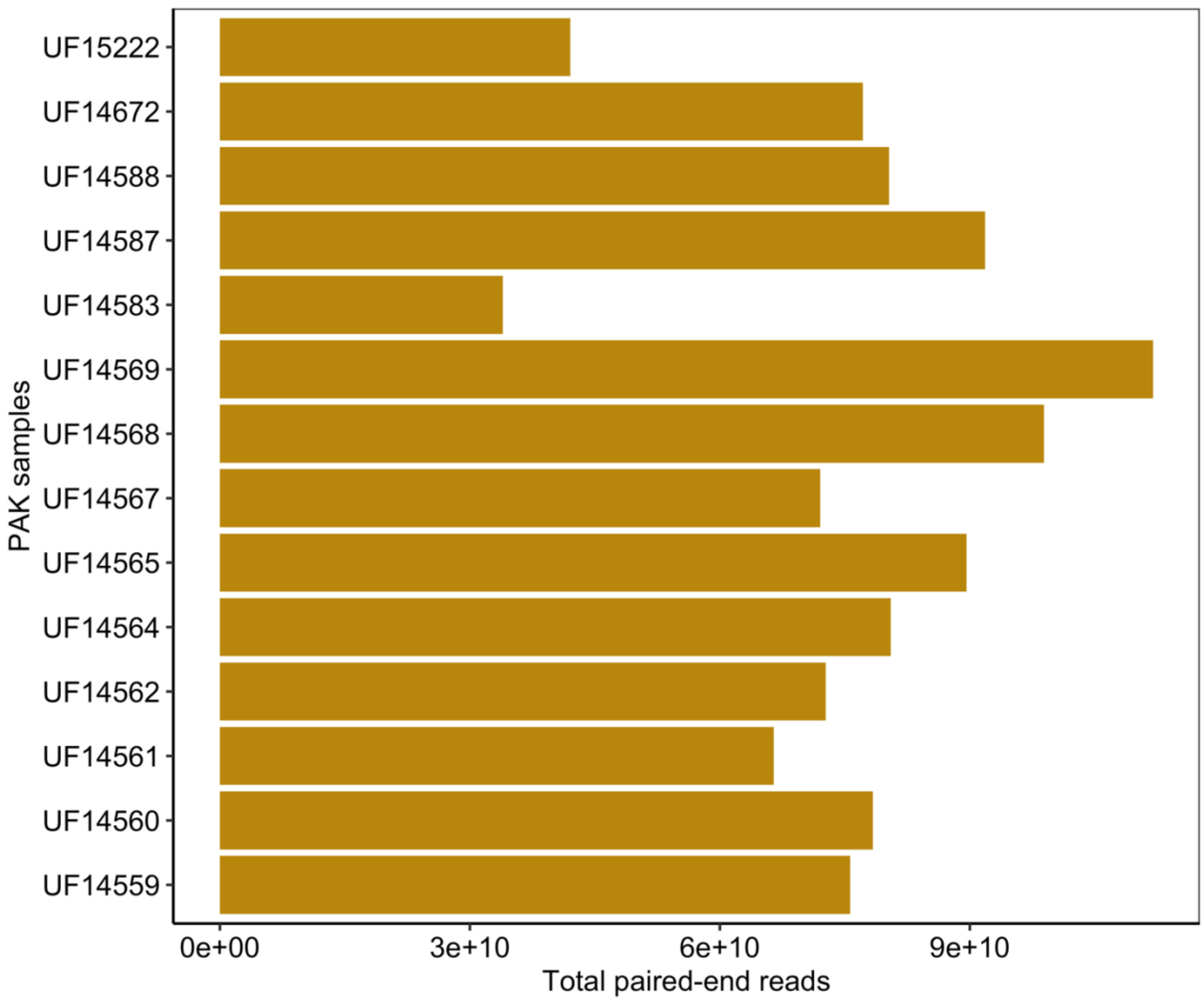

Supplementary Figure S3: Bar plot of the number of paired-end reads generated for each PAK sample.
